# Antioxidant, Antibacterial, and Cytotoxic Activities of the Ethanolic* Origanum vulgare* Extract and Its Major Constituents

**DOI:** 10.1155/2016/1404505

**Published:** 2016-03-09

**Authors:** John Coccimiglio, Misagh Alipour, Zi-Hua Jiang, Christine Gottardo, Zacharias Suntres

**Affiliations:** ^1^Department of Biology, Lakehead University, 955 Oliver Road, Thunder Bay, ON, Canada P7B 5E1; ^2^Faculty of Medicine and Dentistry, Department of Pediatrics, University of Alberta, Edmonton, AB, Canada; ^3^Department of Chemistry, Lakehead University, 955 Oliver Road, Thunder Bay, ON, Canada P7B 5E1; ^4^Medical Sciences Division, Northern Ontario School of Medicine, Lakehead University, 955 Oliver Road, Thunder Bay, ON, Canada P7B 5E1

## Abstract

Oregano is a perennial shrub that grows in the mountains of the Mediterranean and Euro/Irano-Siberian regions. This study was conducted to identify the major constituents of the ethanolic* Origanum vulgare* extract and examine the cytotoxic, antioxidant, and antibacterial properties of the extract but more importantly the contribution of its specific major constituent(s) or their combination to the overall extract biological activity. Gas chromatography/mass spectroscopy analysis showed that the extract contained monoterpene hydrocarbons and phenolic compounds, the major ones being carvacrol and thymol and to a lesser extent p-cymene, 1-octacosanol, creosol, and phytol. A549 epithelial cells challenged with the extract showed a concentration-dependent increase in cytotoxicity. A combination of thymol and carvacrol at equimolar concentrations to those present in the extract was less cytotoxic. The A549 cells pretreated with nonlethal extract concentrations protected against hydrogen-peroxide-induced cytotoxicity, an antioxidant effect more effective than the combination of equimolar concentrations of thymol/carvacrol. Inclusion of p-cymene and/or 1-octacosanol did not alter the synergistic antioxidant effects of the carvacrol/thymol mixture. The extract also exhibited antimicrobial properties against Gram-positive and Gram-negative bacterial strains including clinical isolates. In conclusion, the oregano extract has cytotoxic, antioxidant, and antibacterial activities mostly attributed to carvacrol and thymol.

## 1. Introduction

Herbs and spices contain a wide array of phytochemicals with strong biological and pharmacological properties [1]. One of them, oregano, is a perennial shrub native to the dry, rocky calcareous soils in the mountainous area of the Mediterranean and Euro/Irano-Siberian regions but it is also cultivated for its uses as a herb and therapeutic properties. Studies examining the antioxidant activities of different forms of oregano (fresh, dry, and ready-to-use herb blend pastes) showed that oregano retains its strong antioxidant capacity in both fresh and dry form [2]. The leaves and dried herb of oregano as well as its essential oil are traditionally used for respiratory disorders, indigestion, and rheumatoid arthritis [3–6].

The antibacterial and antioxidant properties of oregano have been attributed mainly to carvacrol and thymol, which are the major components of its essential oil [7]. Antibacterial effects have been reported for oregano against* Clostridium perfringens*,* Pseudomonas aeruginosa*, and* Staphylococcus aureus* [8–10]. Studies comparing the antioxidant properties of Mediterranean food spices and common food additives have shown that extracts from oregano were more effective than butylated hydroxyanisole (BHA) and butylated hydroxytoluene (BHT) in inhibiting lipid peroxidation [11]. The use of synthetic antioxidants to prevent free radical damage can involve questionable nutritional value and toxic side effects while natural antioxidants present in many plants reduce oxidative damage and help in preventing mutagenesis, carcinogenesis, and aging due to their radical scavenging activities [12]. The role of free radicals has been implicated in several pathological conditions, including cancer, cardiovascular diseases, neurodegenerative disorders, and drug toxicity [13–15].

This study was carried out to identify the main components of* Origanum vulgare* from Mt. Parnon, Southern Greece, assess the cytotoxic, antioxidant, and antimicrobial properties of the oregano extract, and delineate the contribution of the extract's major components towards such effects.

## 2. Materials and Methods

### 2.1. Herb Material and Chemicals

The wild-grown herb* Origanum vulgare* was collected and authenticated by Dr. Z. Suntres from the southern slope of Mt. Parnon (37°19′N 22°39′E) in Kynouria Peloponnese, during the early summer of 2009 and voucher specimens are stored in the Department of Chemistry, Lakehead University, Dr. C. Gottardo's laboratory. MTT (3-(4,5-dimethylthiazol-2-yl)-2,5-diphenyltetrazolium bromide) and all other chemicals (i.e., thymol, carvacrol,* p*-cymene, and 1-octacosanol) were purchased from Sigma-Aldrich (Oakville, ON, Canada). The chemicals used were of analytical reagent grade.

### 2.2. Preparation of Extracts

Ground leaves from* Origanum vulgare* (5.5 g) were extracted in 100 mL of ethanol at room temperature for three times, each for 24 h. The combined ethanol solution (300 mL) was concentrated using a rotary evaporator* in vacuo* (at 28°C), resulting in an organic oil (1.04 g, 19% from the dried sample).

### 2.3. Gas Chromatography/Mass Spectrometry (GC-MS)

Two hundred (200) *μ*g of dried extract was dissolved in a 1.1 mL ethanol. The sample and standards (1 *μ*L) were loaded into the Varian 300 GC-MS. The samples were analyzed by a Varian model-450 gas chromatograph coupled with a Varian model 300-MS quadruple GC-MS mass spectrometer. This was attached with a factor four capillary column (VF-5 ms, 30 mm × 0.25 mm ID, DF = 0.25 *μ*m). Helium was used as the carrier gas with a flow rate of 1.0 mL/min. Samples were introduced through a split mode method. This involved a one in ten split by a Varian 450 autosampler, with a high temperature injection port (280°C). The oven temperature was initially 50°C for 1 min. This was increased to a final temperature of 280°C at a rate of 10°C/min. The final 280°C temperature was held for 6 min. Electron ionization conditions were used with ionization energy of 70 eV. The scan range was from 70 to 600 amu. Lastly, the GC-MS interface temperature was set to 270°C.

The components were identified based on the comparison of their RI (retention indices) and mass spectra (NIST08 Mass Spectrum Library) of the GC-MS system. Synthetic components (Sigma-Aldrich Co., Oakville, ON, Canada) were used as references for retention time calculations. Major peaks with respective Mass Spectrum Library matches are shown in Table 1. All determinations were performed in triplicate.

### 2.4. Cell Culture

Human alveolar type II-like epithelial A549 cells (American Type Culture Collection, Manassas, VA, USA) were maintained in Corning Costar 0.2 *μ*m vent cap cell culture flasks (Corning, NY, USA) with standard Dulbecco's modified Eagle's medium nutrient mixture F-12 Ham supplemented with 10% iron-fortified bovine calf serum (SAFC Biosciences, Lenexa, KS, USA), 2 mM l-glutamine, and antibiotic/antimycotic (100 U/mL penicillin, 100 *μ*g/mL streptomycin, and 0.25 *μ*g/mL amphotericin B) from Gibco (Carlsbad, CA, USA). Cultures were incubated at 37°C in a humidified atmosphere of 5% CO_2_ in air and subcultured when they were 80% confluent. Prior to plating, cell counts and viabilities were assessed using a Vi-Cell XR Cell Viability Analyzer (Beckman Coulter, Mississauga, ON, Canada).

### 2.5. Cytotoxic and Antioxidant Properties of Oregano Extracts and Major Extract Components

The A549 cells were seeded into sterile flat-bottom 96-well plates at 10,000 cells/well and grown to 80% confluence overnight before treatments began. To determine the cytotoxicity of ethanolic extracts and their individual components, cells were treated with the different treatments (thymol, carvacrol, p-cymene, and/or 1-octacosanol dissolved in 1% ethanol [the vehicle solution had no effect on the antioxidant and cytotoxic activities of the extract and/or its constituents]) in media. To determine the antioxidant effects of the oregano extracts and their individual components (thymol carvacrol, p-cymene, and/or 1-octacosanol dissolved in 1% ethanol), A549 cells were first pretreated for 24 h with the different treatments in media, followed by treatment with 500 *μ*M hydrogen peroxide. The MTT assay, a commonly used measure of cell viability, was used to assess cell survival as described by the manufacturer (Sigma-Aldrich Co., Oakville, ON, Canada). Viabilities of challenged cells were assessed relatively to control cells.

### 2.6. Bacterial Strains

*P. aeruginosa* (ATCC 25619 and clinical isolates),* Bordetella bronchiseptica* (ATCC 4617, ATCC 10580),* Escherichia coli* (ATCC 25922, ATCC 700973),* Burkholderia cenocepacia *(ATCC 25608 and clinical isolates),* Acinetobacter lwoffii* (ATCC 17925),* Acinetobacter baumannii* (ATCC 19606),* Moraxella catarrhalis* (ATCC 8176),* Bacillus subtilis* (ATCC 6633), and* S. aureus* (ATCC 29213 and clinical isolates) were purchased from Cedarlane (Burlington, ON, Canada) or obtained from the Clinical Microbiology Laboratory of Memorial Hospital (Sudbury, ON, Canada). For experimentation, the strains were inoculated onto Mueller Hinton II agar plates and incubated for 24 h at 37°C.

### 2.7. The Minimum Inhibitory Concentrations (MIC) of Oregano Extract against Gram-Positive and Gram-Negative Strains

The inhibitory concentrations were determined by the agar dilution method as previously described [16]. Bacterial inocula were prepared from an overnight culture in Mueller Hinton II agar and adjusted to contain an equivalence of a 0.5 McFarland standard with phosphate-buffered saline. One (1) *μ*L of the adjusted inocula was then delivered onto Mueller Hinton II agar plates containing twofold serial dilutions of oregano from 25000 *μ*g/mL to 3100 *μ*g/mL, using the Replianalyzer system (Oxoid Inc., Nepean, ON, Canada). The lowest concentration of oregano that prevented the appearance of a visible growth within the inoculation area after 24 h at 36°C was defined as the MIC.

### 2.8. Carvacrol and Thymol Cellular Uptake

The cellular uptake of carvacrol and thymol was examined following exposure of A549 cells (3 × 10^6^ cells) to 56 *μ*M carvacrol or 23.3 *μ*M thymol (i.e., equimolar concentrations to those found in the alcoholic extract) or a combination of the two components. Treated cells were removed at 1, 4, 8, 12, and 24 h, washed twice with PBS, and lysed with NP-40 lysis buffer. Cytosolic and membrane fractions were separated via centrifugation at 10,000 rpm for 12 minutes. Membrane and cytosolic fractions were dissolved in diethyl ether, evacuated with nitrogen gas, capped, and crimped. The samples were analyzed by gas chromatography/mass spectrometry (GC-MS) as previously described.

### 2.9. Statistical Analysis

All data were expressed as means ± SD of triplicate measurements. Data were evaluated by one way analysis of variance (ANOVA). If the *F* values were significant, Student's *t*-test was used to compare all groups. The level of significance was accepted at *p* < 0.05.

## 3. Results

### 3.1. GC-MS Analysis

In the present study, the GC-MS analysis of the ethanol extract of wild-growing herb* Origanum vulgare* obtained from Southern Greece revealed an abundance of monoterpene hydrocarbons and phenolic compounds with the main constituents being carvacrol (2-methyl-5-(1-methylethyl)phenol) (59.46%), followed by thymol (5-methyl-2-(1-methylethyl)phenol) (25.00%),* p*-cymene (6.90%), and 1-octacosanol (4.05%) (Table 1).

### 3.2. Chemotherapeutic Properties of the Oregano Components

**Figure 1:**
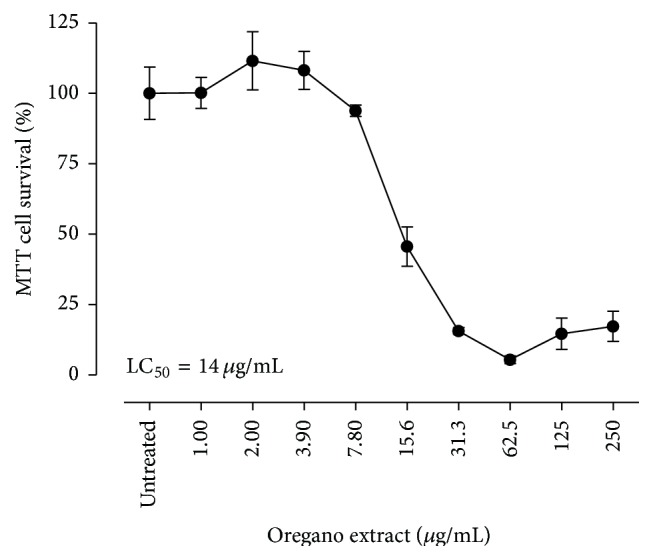
The cytotoxic properties of the ethanolic* O. vulgare* extract in A549 cells. All experiments were repeated three times in two replicates as outlined in Section 2.

In order to examine the chemotherapeutic properties of the oregano extracts, A549 human lung adenocarcinoma epithelial cells were treated with increasing concentrations of oregano ethanolic extracts (0–250 *μ*g/mL final concentration) and cell viability was assessed 24 h after treatment. Treatment of A549 cells with oregano extract resulted in a concentration-dependent decrease in cell viability with a calculated LC_50_ = 14 *μ*g/mL (Figure 1).

**Figure 2:**
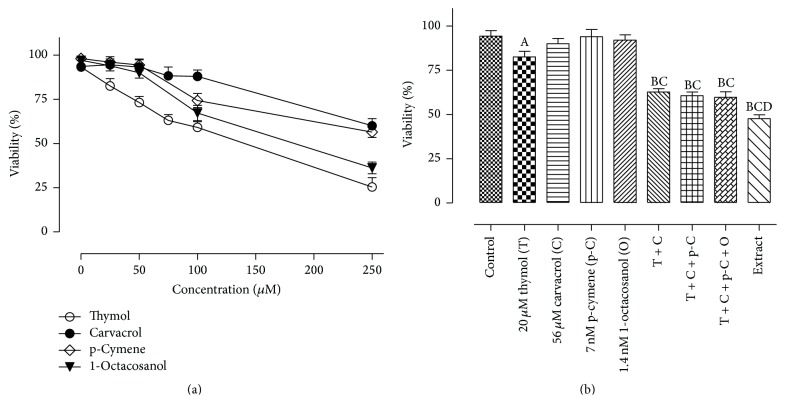
The cytotoxic effects of carvacrol, thymol, p-cymene, and 1-octacosanol in A549 cells. All experiments were repeated three times in two replicates as outlined in Section 2. The thymol and carvacrol concentrations are equimolar to those present in the ethanolic extract. (A) Significantly different from control, *p* < 0.05; (B) significantly different from thymol-treated group, *p* < 0.05; (C) significantly different from carvacrol-treated group, *p* < 0.05; (D) significantly different from carvacrol + thymol + p-cymene + 1-octacosanol-treated group, *p* < 0.05.

To assess the contribution of the major components isolated from the oregano extract on cytotoxicity, the cell viability of A549 cells challenged with thymol, carvacrol, p-cymene, 1-octacosanol, or a mixture containing all four major constituents was examined. As shown in Figure 2(a), challenge of A549 cells with increasing concentrations of thymol, carvacrol, p-cymene, or 1-octacosanol alone resulted in a concentration-dependent decrease in cell viability, with thymol being the most cytotoxic. Challenge of A549 cells with a combination of thymol, carvacrol,* p*-cymene, and 1-octacosanol at equimolar concentrations to the extract (56 *μ*M carvacrol, 23.3 *μ*M thymol, 7.19 nM* p*-cymene, and 1.38 nM 1-octacosanol) was less cytotoxic than the extract itself (% viability of mixture versus oregano extract: 62.65 ± 1.97 versus 49.59 ± 2.26). It should be noted that the contribution of* p*-cymene and 1-octacosanol to the cytotoxicity of the mixture was negligible (Figure 2(b)).

### 3.3. Carvacrol and Thymol Cellular Uptake

**Figure 3:**
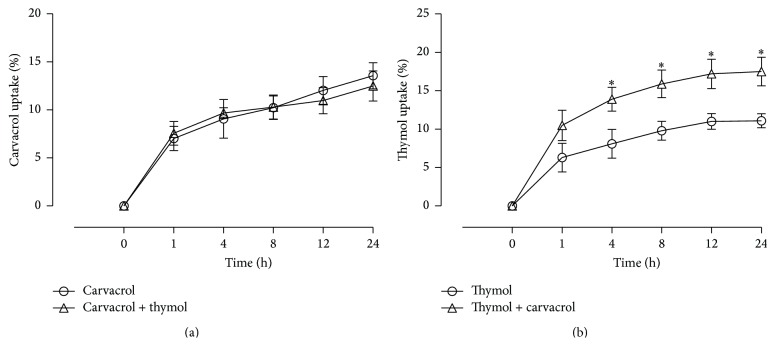
Uptake of carvacrol and thymol in A549 cells. All experiments were repeated three times in two replicates as outlined in Section 2. *∗* denotes significant difference from thymol-treated group, *p* < 0.05.

To investigate the synergistic effect of carvacrol and thymol cytotoxicity, the cellular uptake of carvacrol and thymol was examined (note: 56 *μ*M carvacrol resulted in cell viability of 94.20 ± 2.56% while 23.3 *μ*M thymol resulted in 82.60 ± 2.12% and their combination resulted in 62.65 ± 1.97% viability). Incubation of cells with carvacrol or thymol alone resulted in a time-dependent increase in their uptake (Figures 3(a) and 3(b)). Coincubation of cells with a mixture of carvacrol and thymol did not have any effect on the uptake of carvacrol (Figure 3(a)); a small but significant increase in the uptake of thymol was observed (Figure 3(b)).

### 3.4. Antioxidant Protective Effects of the Oregano Extract

**Figure 4:**
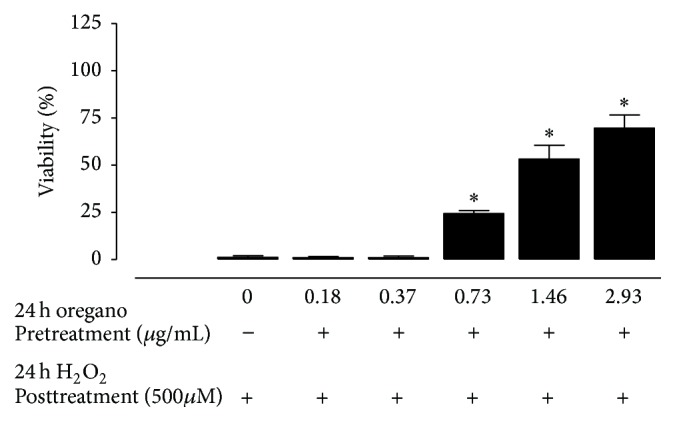
The antioxidant effect of the ethanolic* O. vulgare* extract against H_2_O_2_-induced cytotoxicity in A549 cells. All experiments were repeated three times in two replicates as outlined in Section 2. *∗* denotes significance from the control group treated with H_2_O_2_ only, *p* < 0.05.

**Figure 5:**
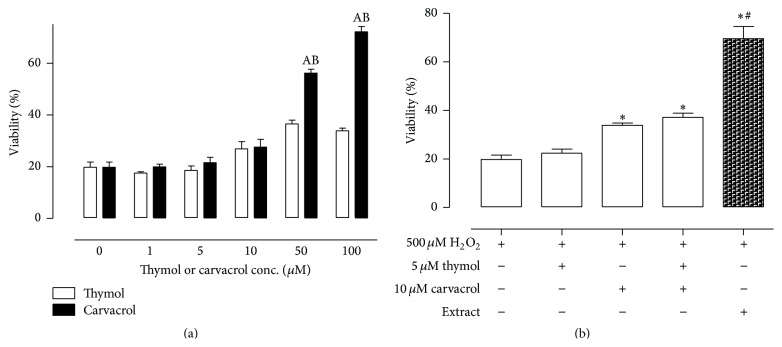
The antioxidant effect of (a) carvacrol and thymol and (b) thymol and/or carvacrol. Equimolar concentration to carvacrol and thymol is present in the extract at the 2.93 *μ*g/mL concentration, as well as in ethanolic extract against H_2_O_2_-induced cytotoxicity in A549 cells. All experiments were repeated three times in two replicates as outlined in Section 2. (A) denotes significance from the control group treated with H_2_O_2_ only, *p* < 0.05; (B) denotes significance from the thymol pretreated group challenged with H_2_O_2_, *p* < 0.05; *∗* denotes significance from the control group treated with H_2_O_2_ only, *p* < 0.05; # denotes significance from the control group pretreated with thymol and carvacrol mixture and challenged with H_2_O_2_, *p* < 0.05.

To examine the antioxidant effects of the oregano extract, noncytotoxic concentrations of extract that corresponded to greater than 95% cell viability were used. As shown in Figure 4, pretreatment of A549 cells with the oregano extract at concentrations ranging from 0 to 2.93 *μ*g/mL resulted in a concentration-dependent protective effect against H_2_O_2_. Preincubation of A549 cells with nontoxic concentrations of carvacrol or thymol showed that both phenolic isomers protected against hydrogen-peroxide induced cytotoxicity, with carvacrol producing a better protective effect than thymol (Figure 5(a)). Preincubation of cells with a combination of both isomers at equimolar concentrations (12 *μ*M carvacrol and 5 *μ*M thymol) to those present in the oregano extract (at the 2.93 *μ*g/mL concentration) resulted in a less protective effect than that produced by the extract (% viability of carvacrol + thymol mixture versus oregano extract: 37.14 ± 0.77 versus 69.60 ± 5.00) (Figure 5). Inclusion of p-cymene and/or 1-octacosanol to the carvacrol and thymol mixture did not contribute to the antioxidant effects of the mixture (data not shown); however, the inclusion of the other oregano extracts to the carvacrol/thymol mixture was not examined.

### 3.5. Antimicrobial Properties of the Oregano Extract

The antibacterial properties of the ethanolic oregano extract were measured using an agar dilution method. The oregano extract inhibited the growth of reference ATCC Gram-negative and Gram-positive bacterial strains with varying degree, but no trends were realised (Table 2). The inhibitory concentrations were 2- to 4-fold lower against nonmucoid than mucoid clinical isolates of* P. aeruginosa* and exhibited greater effects against clinical isolates of* B. cenocepacia* compared to the ATCC reference strain (Table 2).

## 4. Discussion

Results from the GC-MS analysis of the ethanol extract of wild-growing herb* Origanum vulgare* obtained from Southern Greece revealed an abundance of monoterpene hydrocarbons and phenolic compounds with the main constituents being carvacrol and thymol (Table 1). This finding is consistent with that reported by other investigators who found that in spite of the high variability of individual compounds in the essential oil of* O. vulgare* from 23 localities scattered all over Greece, the sum of carvacrol, thymol,* p*-cymene, and *γ*-terpinene was consistent, amounting to >80% [10, 17, 18]. Plants collected from the northern region of Greece were rich in thymol (30.3–42.8% of total oil), whereas those from the southern part of the country were rich in carvacrol (57.4–69.6% of total oil) [17].* p*-Cymene (1-methyl-4-(1-methylethyl)-benzene) and *γ*-terpinene (1-methyl-4-(1-methylethyl)-1,4-cyclohexadiene) are the precursors of carvacrol and thymol in species of* Origanum* [19].

Studies examining the effect of oregano extract on cancer prevention and cytotoxicity are limited [20]. In the present study, challenge of A549 human lung adenocarcinoma epithelial cells with oregano ethanolic extracts (0–250 *μ*g/mL final concentration) resulted in a concentration-dependent decrease in cell viability with a calculated LC_50_ = 14 *μ*g/mL (Figure 1). It is not known whether the cytotoxicity of oregano extract is attributed to a specific component or combination of components. Results shown in Figure 2 demonstrated that challenge of A549 cells with thymol, carvacrol, p-cymene, or 1-octacosanol alone resulted in a concentration-dependent decrease in cell viability, with thymol being more cytotoxic than the other three compounds. Challenge of A549 cells with a combination of the four compounds at equimolar concentrations (56 *μ*M carvacrol, 23.3 *μ*M thymol) to those present in the oregano extract was less cytotoxic than the extract itself. The cytotoxicity of the oregano extract is mostly attributed to presence of carvacrol and thymol.* p*-cymene and 1-octacosanol, although cytotoxic at high concentrations, did not contribute to the cytotoxicity of the mixture, most likely attributed to their low potency and very low availability in the mixture (nM range). It appears that other unmeasured ethanolic constituents at lower concentrations than those determined in this study might possess higher potencies and play a role in the overall effects of the oregano extracts.

The mechanisms by which thymol and carvacrol cause cell death in mammalian cell lines have not been thoroughly investigated. Results from an* in vitro* study showed that carvacrol is very potent inhibitor of cell growth in A549 cell line as evidenced by the concentration-dependent decreases in cell number, degeneration of cell morphology, and a decrease in total protein amount [21]. Thymol induces cell death in human osteosarcoma and astrocytes and may involve apoptosis via mitochondrial pathways [22, 23]. Whether the synergistic effects of carvacrol and thymol regarding cell viability are related to a combination of the purported mechanisms is under investigation. It is evident from the results of this study that coincubation of carvacrol and thymol increased the uptake of the more cytotoxic thymol and enhanced the cytotoxicity of the mixture. Carvacrol is slightly more lipophilic than thymol with partition coefficient in octanol/water (*P*_o/w_) of 3.64 and 3.30, respectively [24–26], suggesting that carvacrol is partitioned deeper in the cytoplasmic membrane, thereby causing an expansion of the membrane [24] altering its permeability [27].

Oxidative stress describes the outcome of an increased reactive oxygen species (ROS) production and/or a decrease in their elimination. ROS (e.g., superoxide anion, hydrogen peroxide, hydroxyl radical, and lipid peroxides) can be formed as normal products of aerobic metabolism but can be produced at elevated rates under pathophysiological conditions. ROS attack biological macromolecules such as membrane lipids, nucleic acids, carbohydrates, and proteins resulting in damage [13–15, 28]. Pretreatment of A549 cells with the oregano extract at nontoxic concentrations ranging from 0 to 2.93 *μ*g/mL resulted in a concentration-dependent protective effect against H_2_O_2_. H_2_O_2_ has been used as a model of oxidative stress because it can be generated* in vivo* by the spontaneous and/or enzymatic dismutation of the superoxide anion radical. The antioxidant effectiveness of oregano extracts* in vitro* is perhaps due to their ability to act as reducing agents and free radical scavengers, as quenchers of singlet O_2_ formation and to complex with prooxidant metal ions [29, 30]. Phenols (thymol, carvacrol) monocyclic hydrocarbons (terpinolene, R-terpinene, and*γ*-terpinene) belong to the most active natural antioxidants found in the essential oils [29, 30]. Indeed, in our study nontoxic concentrations of both phenolic isomers protected against hydrogen-peroxide induced cytotoxicity, with carvacrol producing a better protective effect than thymol. However, a combination of both isomers at equimolar concentrations (12 *μ*M carvacrol and 5 *μ*M thymol) to those present in the oregano extract (at the 2.93 *μ*g/mL concentration) resulted in a less protective effect than that produced by the extract (Figure 4). Although inclusion of* p*-cymene and/or 1-octacosanol to the carvacrol and thymol mixture did not contribute to the antioxidant effects of the mixture, other components present in the extract at lower levels might possess higher antioxidant potencies and play a role in the overall effects of the oregano extracts. For example, *β*-caryophyllene, a constituent in oregano at very low levels, has a higher inhibitory capacity on lipid peroxidation than probucol, *α*-humulene, and *α*-tocopherol [31, 32].

Gram-negative pathogens like* P. aeruginosa* and* B. cenocepacia* are commonly found opportunistic bacilli in our environment that establish infections in patients suffering from pulmonary diseases like cystic fibrosis [33, 34]. The results of this study showed that the ethanolic extracts of oregano exhibited antibacterial properties as indicated by their ability to inhibit nonmucoid and mucoid clinical isolates of* P. aeruginosa*, and clinical isolates of* B. cenocepacia*. It is important to note that the extracts were less effective against the mucoid clinical isolates of* P. aeruginosa* possibly due to the secretion of negatively charged alginate-rich matrix to the surroundings, which inhibit antibiotic penetration. Earlier work by Lambert et al. [8] credits the activity of oregano essential oil and its active constituents (carvacrol and thymol) to interference with the pH gradient and membrane permeability. Burt et al. [35] have previously described that carvacrol may also be involved in inhibiting* E. coli* flagellin. A study examining the separate and combined antibacterial activities of the main chemical constituents of oregano and other spices (namely, eugenol, cinnamaldehyde, thymol, and carvacrol) showed that each component possessed antibacterial properties and the components acted synergistically with each other [8, 36]. The mechanism by which the oregano extract and individual components produce the antimicrobial effect on mucoid and nonmucoid bacteria is currently under investigation.

In conclusion, the results of this study showed that the ethanolic extract of* Origanum vulgare* possesses strong cytotoxic, antioxidant, and antibacterial activities which are attributed mostly to the presence of the isomeric phenolic constituents, carvacrol, and thymol.

## Figures and Tables

**Table 1:** Composition of *Origanum vulgare* assessed by GC-MS analysis.

Peak	Retention time (min)	% area	Compound
1	8.959	6.900	1-Methyl-4-(1-methylethyl) benzene- (p-cumene)
2	9.664	1.904	1-Methyl-4-(1-methylethyl)-1,4-cyclohexadiene (*γ*-terpinene)
3	13.113	2.110	1-Methoxy-4-methyl-2-(1-methylethyl) benzene (creosol)
4	14.072	25.008	2-(1-Methylethyl)-5-methylphenol (thymol)
5	14.263	59.468	2-Methyl-5-(1-methylethyl)-phenol (carvacrol)
6	19.327	0.560	3,7,11,15-Tetramethyl-2-hexadecen-1-ol (phytol)
7	25.600	4.050	1-Octacosanol

**Table 2:** Minimum Inhibitory Concentrations (MIC) of bacterial strains.

Bacterial strains	Source	Ethanolic oregano extract (*μ*g/mL)
*Pseudomonas aeruginosa*		
25619	ATCC	25
Mucoid clinical isolate 1	Cystic fibrosis isolate	25
Mucoid clinical isolate 2	Cystic fibrosis isolate	25
Mucoid clinical isolate 3	Cystic fibrosis isolate	25
Mucoid clinical isolate 4	Cystic fibrosis isolate	25
Mucoid clinical isolate 5	Cystic fibrosis isolate	12.5
Nonmucoid clinical isolate 1	Cystic fibrosis isolate	6.3
Nonmucoid clinical isolate 2	Cystic fibrosis isolate	6.3
Nonmucoid clinical isolate 3	Cystic fibrosis isolate	25
Nonmucoid clinical isolate 4	Cystic fibrosis isolate	12.5
*Bordetella bronchiseptica*		
10580	ATCC	12.5
4617	ATCC	12.5
*Escherichia coli*		
25922	ATCC	25
700973	ATCC	12.5
*Burkholderia cenocepacia*		
25608	ATCC	25
Clinical isolate 1	Cystic fibrosis isolate	12.5
Clinical isolate 2	Cystic fibrosis isolate	6.3
Clinical isolate 3	Cystic fibrosis isolate	12.5
Clinical isolate 4	Cystic fibrosis isolate	12.5
*Acinetobacter lwoffii* 17925	ATCC	12.5
*Acinetobacter baumannii *19606	ATCC	12.5
*Moraxella catarrhalis* 8176	ATCC	12.5
*Bacillus subtilis* 6633	ATCC	6.3
*Staphylococcus aureus *		
29213	ATCC	25
Clinical isolate 1	Cystic fibrosis isolate	12.5
Clinical isolate 2	Cystic fibrosis isolate	25
